# Heart-rate tuned comb filters for processing photoplethysmogram (PPG) signals in pulse oximetry

**DOI:** 10.1007/s10877-020-00539-2

**Published:** 2020-06-17

**Authors:** Ludvik Alkhoury, Ji-won Choi, Chizhong Wang, Arjun Rajasekar, Sayandeep Acharya, Sean Mahoney, Barry S. Shender, Leonid Hrebien, Moshe Kam

**Affiliations:** 1 Department of Electrical and Computer Engineering, Newark College of Engineering, New Jersey Institute of Technology, New Jersey, 07102 USA; 2 Department of Electrical and Computer Engineering, Drexel University, Philadelphia, PA 19104 USA; 3 Regulatory Affairs Department, Athena GTX, Johnston, IA 501131 USA; 4 Human Systems Department, Naval Air Warfare Center Aircraft Division, Patuxent River, MD 20670 USA

**Keywords:** Pulse oximeter, Peripheral capillary oxygen saturation $( {\text{SpO}}_{{\text{2}}} )$, Comb filter, Electrocardiography (ECG), Motion artifact, Pulse oximetry, Photoplethysmography (PPG)

## Abstract

Calculation of peripheral capillary oxygen saturation ${\text{(SpO}}_{{\text{2}}} {\text{)}}$ levels in humans is often made with a pulse oximeter, using photoplethysmography (PPG) waveforms. However, measurements of PPG waveforms are susceptible to motion noise due to subject and sensor movements. In this study, we compare two ${\text{SpO}}_{{\text{2}}}$-level calculation techniques, and measure the effect of pre-filtering by a heart-rate tuned comb peak filter on their performance. These techniques are: (1) “Red over Infrared,” calculating the ratios of AC and DC components of the red and infrared PPG signals,$\frac{(AC/DC)_{red}}{(AC/DC)_{infrared}}$, followed by the use of a calibration curve to determine the ${\text{SpO}}_{{\text{2}}}$ level Webster (in: Design of pulse oximeters, CRC Press, Boca Raton, 1997); and (2) a motion-resistant algorithm which uses the Discrete Saturation Transform (DST) (Goldman in J Clin Monit Comput 16:475–83, 2000). The DST algorithm isolates individual “saturation components” in the optical pathway, which allows separation of components corresponding to the ${\text{SpO}}_{{\text{2}}}$ level from components corresponding to noise and interference, including motion artifacts. The comparison we provide here (employing the two techniques with and without pre-filtering) addresses two aspects: (1) accuracy of the ${\text{SpO}}_{{\text{2}}}$ calculations; and (2) computational complexity. We used both synthetic data and experimental data collected from human subjects. The human subjects were tested at rest and while exercising; while exercising, their measurements were subject to the impacts of motion. Our main conclusion is that if an uninterrupted high-quality heart rate measurement is available, then the “Red over Infrared” approach preceded by a heart-rate tuned comb filter provides the preferred trade-off between ${\text{SpO}}_{{\text{2}}}$-level accuracy and computational complexity. A modest improvement in ${\text{SpO}}_{{\text{2}}}$ estimate accuracy at very low SNR environments may be achieved by switching to the pre-filtered DST-based algorithm (up to 6% improvement in ${\text{SpO}}_{{\text{2}}}$ level accuracy at −10 dB over unfiltered DST algorithm and the filtered “Red over Infrared” approach). However, this improvement comes at a significant computational cost.

## Introduction

**Fig. 1:**
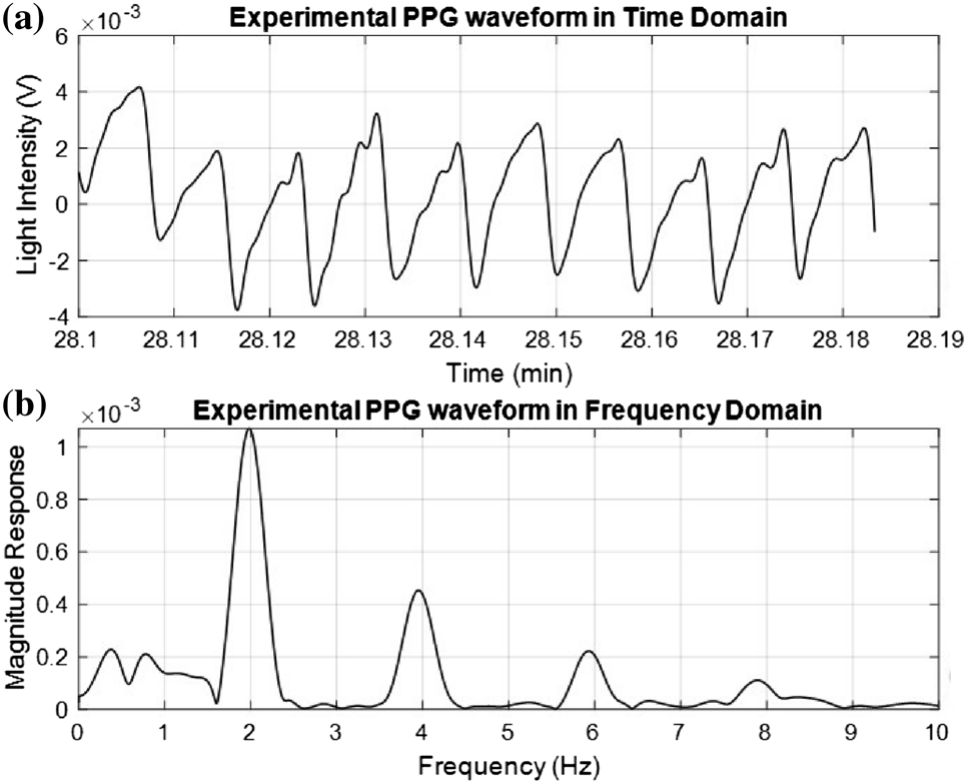
**a** Time domain representation of a PPG signal extracted from experimental data—**b** Frequency domain representation of a PPG signal extracted from experimental data

Photoplethysmography (PPG) is a noninvasive [1, 2], electro-optic method for detecting the cardiovascular pulse wave generated by the elastic nature of the peripheral vascular arteries excited by the quasi-periodic contractions of the heart [3]. Vital signs such as heart rate, respiratory rate, and blood oxygen saturation are usually extracted from PPG waveforms. Fig. 1 illustrates a clean PPG signal obtained from experimental data in the time domain (Fig. 1a) and in the frequency domain (Fig. 1b). This clean PPG signal was taken from a healthy male human in the course of intensive exercise regime.^1^

A pulse oximeter detects and calculates the absorption of light by functional hemoglobin (oxygenated and deoxygenated hemoglobin) to produce a measurement of the peripheral capillary oxygen saturation $( {\text{SpO}}_{{\text{2}}} )$. ${\text{SpO}}_{{\text{2}}}$ is an estimate of arterial oxygen saturation (${\text{SaO}}_{2}$) [4]. The use of absorption of light for calculation of ${\text{SpO}}_{{\text{2}}}$ levels makes use of the Lambert–Beer Law. The Law states that if a solute is dissolved in a clear solvent, its concentration can be determined if a light of known wavelength is transmitted through the solution. The incident and transmitted light intensity are logarithmically related to the absorbance of the solution (the absorbance and the concentration of the solution have a linear relationship) [5]. On average, a healthy human has a ${\text{SpO}}_{{\text{2}}}$ value of 95–100% at sea level. ${\text{SpO}}_{{\text{2}}}$ values below 90% are considered low, and are taken as a possible indication for the onset of hypoxia [6].

In conventional oximetry, ${\text{SpO}}_{{\text{2}}}$ is calculated from the PPG waveforms using the “Red over Infrared” approach, calculating the optical density ratio (ratios of AC and DC components of the red and infrared PPG signals), $r=\frac{(AC/DC)_{red}}{(AC/DC)_{infrared}}$, followed by the use of a calibration curve (see [1], as well as a recent variation in [7]). In most systems, weighted moving average filters are commonly used to stabilize the readings [8–10]. However, when the measured subject experiences substantial motion, measurements become noisier, and the “Red over Infrared” approach sometimes fails, providing false and inaccurate readings [10]. A Discrete Saturation Transform (DST) based algorithm [2] that uses an adaptive noise cancellation filter [11, 12] was proposed to suppress some motion artifact effects on ${\text{SpO}}_{{\text{2}}}$ level calculations, thereby improving pulse oximetry. A 2002 study [13] reviewed the performance of twenty (20) commercial oximeters, and compared ${\text{SpO}}_{{\text{2}}}$ readings from stationary “control hand” of each of the seventy (70) healthy human subjects to readings from the subject’s other hand, which was in motion. In this study, a Masimo SET (Signal Extraction Technology) pulse oximeter, which uses the DST algorithm, exhibited the best performance over all other tested oximeters. Other comparisons of oximeter performances were reported in [14] (from 2016) and [15] (from 2018). The study in [15] also included a DST-based oximeter (Masimo Radical-7). It concluded that in the face of motion artifacts, the DST-based oximeter performed at a similar level to other FDA-cleared pulse oximeters.^2^

In this study we are motivated by the observation that the spectral components of the PPG waveform appear at a fundamental frequency that corresponds to the subject’s heart rate and at its harmonics (e.g., Fig. 1b of this paper and Fig. 7b, d of [16]). A comb filter tuned to these (possibly time-varying) frequencies thus may have the potential to “clean up” the PPG waveform prior to applying the ${\text{SpO}}_{{\text{2}}}$ calculation algorithm. The low computational complexity of a comb filter (when realized in software) may offer a viable alternative to the use of the more computationally complex realization of DST algorithm based systems.

**Fig. 2:**
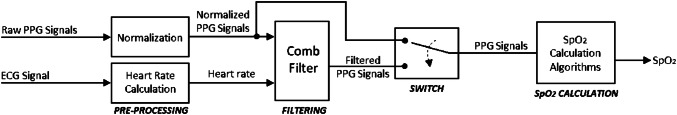
$${\text{SpO}}_{{\text{2}}}$$ Calculation procedure

The rest of the paper is organized as follows. Section 2 reviews two popular methods for ${\text{SpO}}_{{\text{2}}}$ calculations from PPG waveforms, namely, the “Red over Infrared” approach and a DST-based algorithm. Section 3 reviews the synthetic and experimentally measured PPG waveforms used to assess ${\text{SpO}}_{{\text{2}}}$ calculations in this study. Section 4 compares the performance (accuracy and computational complexity) of two ${\text{SpO}}_{{\text{2}}}$ calculation methods, namely, the “Red over Infrared” approach and a DST-based algorithm, on synthetic data and on data collected from human subjects. In that section we also quantify the effect of pre-filtering of the PPG signals with a heart-rate tuned comb filter on algorithm performance. The main conclusion is that if an uninterrupted high-quality heart rate measurement is available, then the pre-filtered “Red over Infrared” approach using a heart-rate tuned comb filter provides the preferred trade-off between ${\text{SpO}}_{{\text{2}}}$ level accuracy and computational complexity. While in a very low signal to noise (SNR) environment, the DST-based algorithm performed somewhat better (up to 6% improvement in accuracy at −10 dB SNR over unfiltered DST algorithm and the filtered “Red over Infrared” approach), its computational complexity was much higher.

## Methods

A block diagram of a processing module for PPG signals towards ${\text{SpO}}_{{\text{2}}}$ level calculation is shown in Fig. 2. The module is subdivided into three main stages: (1) pre-processing, (2) filtering, and (3) ${\text{SpO}}_{{\text{2}}}$ calculation. The inputs are raw PPG (red and infrared signals) and ECG waveforms, and the outputs are ${\text{SpO}}_{{\text{2}}}$ levels. The virtual switch enables comparison of the performance of the ${\text{SpO}}_{{\text{2}}}$ calculation module with and without the comb filter.

**Fig. 3:**
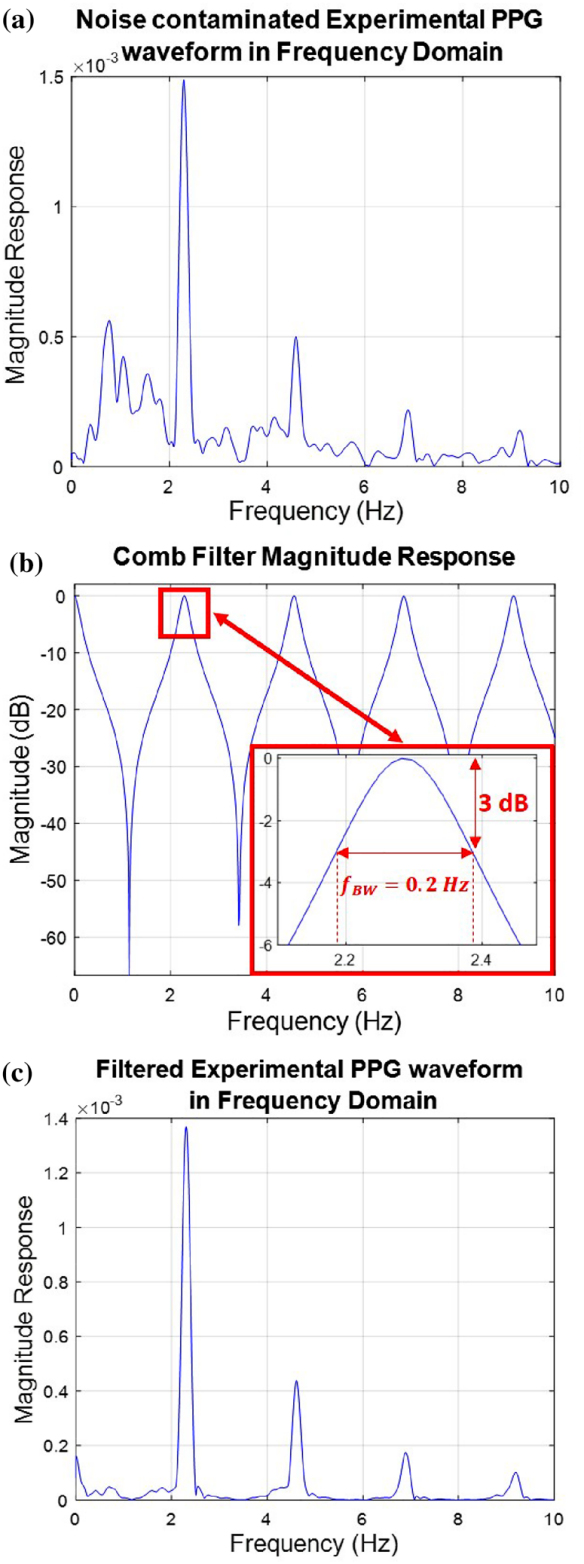
**a** Frequency domain representation of a noise contaminated experimental PPG waveform of fundamental frequency $f_{0}$ = 2.29 Hz—**b** Magnitude response of a tuned comb filter—**c** Frequency domain representation of the comb-filtered PPG waveform

1. In the pre-processing stage, raw PPG signals are normalized (Sect. 2.1). Concurrently, the heart rate (HR) is calculated from an electrocardiography (ECG) waveform which is assumed to be available (Sect. 2.2).
2. In the filtering stage, the normalized PPG waveforms are processed with a heart-rate tuned peak comb filter (Fig. 3b) that uses the calculated HR as a reference signal (Sect. 2.3). The filter presents its lowest attenuation at the heart rate frequency and its principal harmonics, and higher attenuation otherwise.
3. The virtual switch (Fig. 2) allows us to compare the ${\text{SpO}}_{{\text{2}}}$ estimate that uses the normalized PPG signals to the estimate that uses these signals after comb filtering.
4. In the ${\text{SpO}}_{{\text{2}}}$ calculation stage, we use one of two different algorithms “Red over Infrared” [10] or a DST-based algorithm [2].

### Normalization

Raw PPG (red and infrared) waveforms have two main components, namely: an AC component due to the light absorbed by pulsatile arterial blood, and a DC component due to the light absorbed by non-pulsatile components, such as tissues, venous, and capillary blood [17]. Since the DC component varies from one person to another (depending on variables such as skin tone and tissue thickness), a normalization process is commonly used. The normalization is done by dividing the signal’s AC component by its constant DC component.

### Heart rate calculation

We assume that we have access to the ECG waveform of the subject whose ${\text{SpO}}_{{\text{2}}}$ level we measure. The ECG waveform is known to be less susceptible to motion noise than the PPG waveform [18, 19]. The heart rate (beats per minute) was calculated in our study from an ECG signal through the Pan and Tompkins algorithm [20]. Since the fundamental frequency of the PPG signal is the heart rate, we use the heart rate to tune the comb filter. The comb filter discriminates against the portion of the PPG input signals which are not at the heart rate frequency or one of its principal harmonics.

### Comb filter

**Fig. 4:**
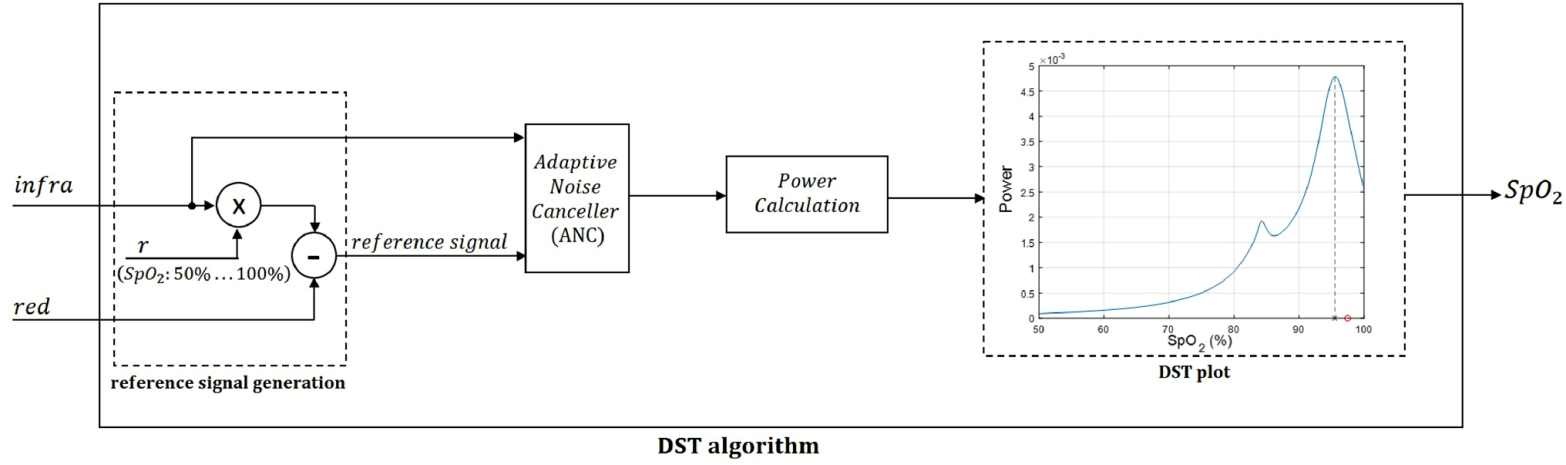
DST algorithm block diagram and DST plot for a noise-contaminated synthetic PPG signal of SNR of 0 dB. The ${\text{SpO}}_{{\text{2}}}$ level calculated by the algorithm corresponds to the right-most peak in the DST plot (output power vs. ${\text{SpO}}_{{\text{2}}}$ level)

The spectral components of the PPG waveform appear at a fundamental frequency (corresponding to the subject’s heart rate) and its harmonics (Fig. 1b of this paper and Fig. 7b, d of [16]). The use of a comb peak filter tuned to these frequencies may therefore serve to clean up the PPG waveform. The filter exhibits low attenuation at the fundamental frequency and its harmonics, and high attenuation in the intermediate regions between these frequencies (see Fig. 3b). In this manner, the filter reduces noise that resides in the intermediate regions. In order to reject as much noise as possible, we want the ‘peaks’ of the filter to be narrow. On the other hand, overly narrow peaks are likely to miss the PPG harmonics if the tuning is not exact (if the filter is not tuned exactly to the heart rate) (Fig. 4). Therefore, a compromise is needed between tuning accuracy and noise-rejection capability. We employed an IIR comb filter with the transfer function

1 $$\begin{aligned} H(z) = \beta \frac{1+z^{-k}}{1-\alpha z^{-k}} , \end{aligned}$$

where $\alpha$ and $\beta$ are two positive scalars and *K* is the comb filter’s order. The design equations are shown in Table 1.

**Table 1:** Comb filter design equations

Comb filter design equations	
$\displaystyle { K=\frac{f_s}{f_0}}\;{\text{(dimensionless)}}$ (dimensionless)	$f_0$ is the fundamental frequency (heart rate) in Hz. $f_s$ is the sampling frequency
$\beta = \frac{{1 - \alpha }}{2}\;{\text{(dimensionless)}}$	Gain at fundamental frequency and its harmonics set to 1
$f_{{BW}} = cos^{{ - 1}} \left( {\frac{{2\alpha }}{{\alpha ^{2} + 1}}} \right) \times \frac{{f_{0} }}{{180}}\;{\text{(Hz)}}$	3 dB bandwidth set

We have selected $f_{BW}=0.2$ Hz (capturing 97.5% of the total power of the signal of interest). For this selection and $f_{0}=1$ Hz, the null-to-null bandwidth of the filter’s lobes is 1 Hz and the 10 dB bandwidth is 0.49 Hz. The sampling rate was $f_{s}=256\,\hbox {Hz}$.

Figure 3 shows a frequency domain plot of a noise contaminated PPG signal measured on a healthy male subject during aerobic exercise (see Sect. 3.2 for more details). The signal is passed through a heart-rate tuned comb filter whose transfer function (magnitude response) is shown in Fig. 3b. The fundamental frequency (frequency of the heart rate) of the subject is $f_{0}=2.29\,\hbox {Hz}$. The parameters of the comb filter are $K=112, \alpha =0.7570$, and $\beta =0.1215$. Fig. 3c shows the clean PPG signal emerging from the comb filter.

### ${\text{SpO}}_{{\text{2}}}$ level calculations

#### The “red over infrared” approach

In the “Red over Infrared” approach, two light sources of different wavelengths, $\lambda _r$ and $\lambda _{ir}$ (red and infrared light, respectively), are used. The optical density ratio ‘r’ is defined as the ratio of the normalized red to the normalized infrared waveforms. In our project, we have used the Texas Instruments AFE4490 as the analog front-end for the pulse oximetry system—using diodes of wavelength of $\lambda _{r} = 660\,\hbox {nm}$ for the red light source and $\lambda _{ir} = 900\,\hbox {nm}$ for the infrared light source. In order to calculate ${\text{SpO}}_{{\text{2}}}$ levels, we employed first the calibration curve (2) which was provided by the manufacturer as the standard model [21].

2 $$\begin{aligned} {\text{SpO}}_{{\text{2}}} = 110 - 25\times r. \end{aligned}$$

**Fig. 5:**
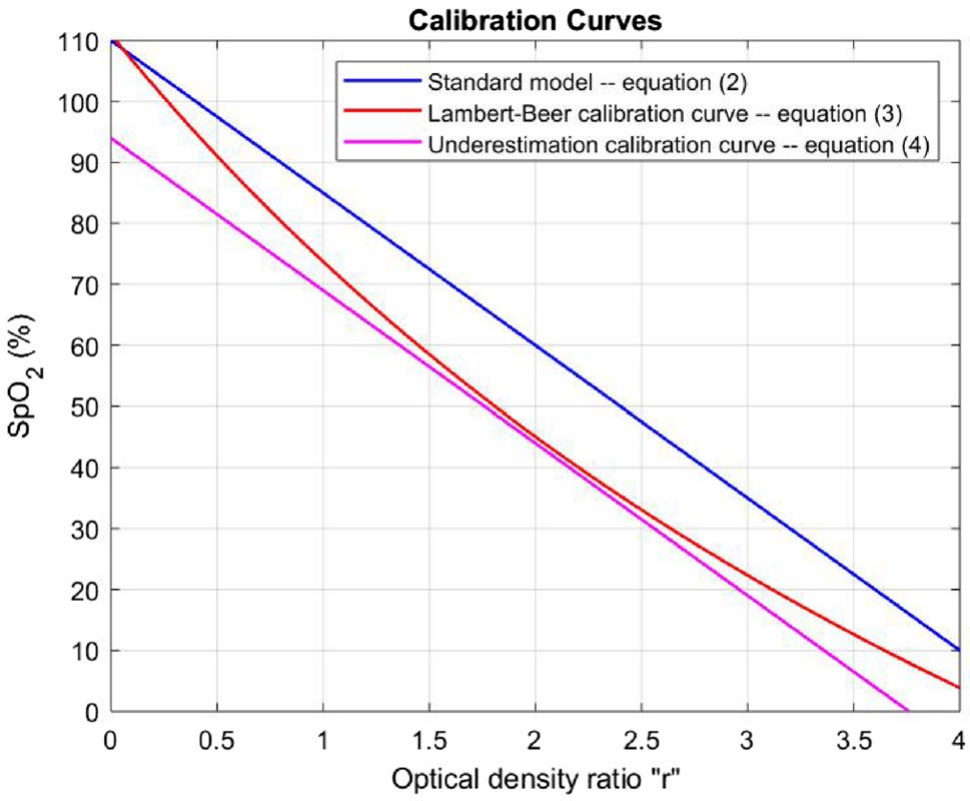
Calibration curves used for sensitivity study

To study the sensitivity of our statistical results and main conclusions (Sect. 4.2 and Table 4) to the specification of the calibration curve, we have also employed two alternate calibrations curves in this study (viz., we calculated the statistics separately for each one of three different calibration curves, see Fig. 5).

The first alternate curve is provided by the Lambert-Beer method [22], shown in Fig. 5 as a red trace. Notably, the standard model (blue trace) shows a relationship between ${\text{SpO}}_{{\text{2}}}$ and ‘r’ which is “to the right and above” Lambert–Beer curve (3). Hence, the standard model overestimates the ${\text{SpO}}_{{\text{2}}}$ level when compared ot the Lambert–Beer estimate at the same value of ‘r’.

The second alternate curve is “to the left and below” the Lambert–Beer curve, and hence underestimates the ${\text{SpO}}_{{\text{2}}}$ level when compared to the Lambert-Beer estimates. We denote this curve (4) “underestimation calibration curve” (it has the same slope (− 25) as the standard model). The equations of the alternate calibration curves are as follows.

Lambert–Beer estimation calibration curve:

3 $${\text{SpO}}_{{\text{2}}} = \frac{{100(r \times Ext({\text{Hb}},\lambda _{{ir}} ) - Ext({\text{Hb}},\lambda _{r} ))}}{{r(Ext({\text{Hb}},\lambda _{{ir}} ) - Ext({\text{HbO}}_{2} ,\lambda _{{ir}} )) + Ext({\text{HbO}}_{2} ,\lambda _{r} ) - Ext({\text{Hb}},\lambda _{r} )}},$$

where $Ext({\text{Hb}},\lambda _{r} )$ and $Ext({\text{Hb}},\lambda _{ir})$ are the extinction coefficients of deoxygenated hemoglobin, and *Ext*( ${\text{HbO}}_{2}$ $,\lambda _{r})$ and $Ext( {\text{HbO}}_{2} ,\lambda _{ir})$ are the extinction coefficients of oxygenated hemoglobin, for wavelengths $\lambda _{r}$ and $\lambda _{ir}$, respectively. In this study, the following extinction coefficients were taken from [23]:

$$Ext({\text{Hb}},\lambda _{r} ) = 3226.6\;{\text{cm}}^{{ - 1}} ({\text{moles/l}})^{{ - 1}}$$ ,

$$Ext({\text{Hb}},\lambda _{ir}) = 761.84\; {\text{cm}}^{{ - 1}} ({\text{moles/l}})^{{ - 1}}$$ ,

$$Ext( {\text{HbO}}_{2} ,\lambda _{r}) = 319.6\; {\text{cm}}^{{ - 1}} ({\text{moles/l}})^{{ - 1}}$$ ,

$$Ext( {\text{HbO}}_{2} ,\lambda _{ir}) = 1198\; {\text{cm}}^{{ - 1}} ({\text{moles/l}})^{{ - 1}}$$ .

Underestimation calibration curve:

4 $${\text{SpO}}_{{\text{2}}} = 94 - 25 \times r.$$

#### The DST algorithm

The DST algorithm [2] was derived to measure ${\text{SpO}}_{{\text{2}}}$ levels in the face of motion noise. In developing the algorithm, it was assumed that the clean PPG signal of interest is contaminated by additive noise, uncorrelated with the signal. Figure 4 is the block diagram of the DST algorithm. The red and infrared PPG signals are the inputs and the ${\text{SpO}}_{{\text{2}}}$ level is the output. A family of reference signals is generated for each optical density ratio corresponding to ${\text{SpO}}_{{\text{2}}}$ values ranging from 50 to 100% at a resolution of 0.5%. The reference signal is defined as

5 $$reference\ signal (t) = infra(t)\times r - red(t).$$

Here, ‘r’ is an arbitrary optical density ratio value that corresponds to ${\text{SpO}}_{{\text{2}}}$ levels ranging from 50% to 100% (we use the calibration curve Eq. (5), which gives the corresponding values of ‘r’ of 0.4 to 2.4). “red(t)” and “infra(t)” are the time-dependent red and infrared PPG signals collected on a range of t $\in$ [0, T] (T is typically 10 s); they serve as the two inputs of the DST algorithm.

**Fig. 6:**
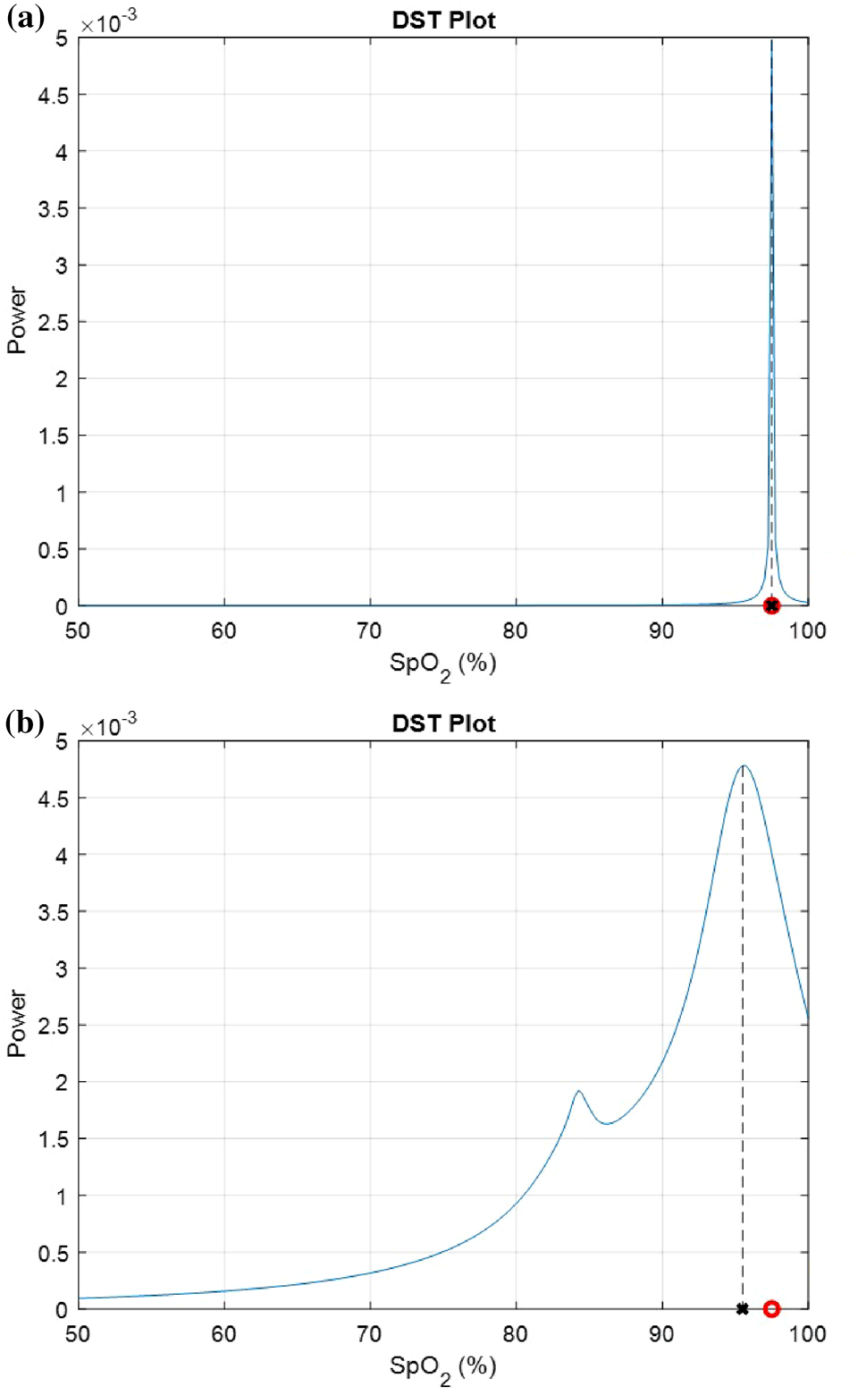
**a** DST plot on a clean synthetic PPG signal—**b** DST plot on noise-contaminated synthetic PPG signals (SNR = 0 dB). The red circle is the ${\text{SpO}}_{{\text{2}}}$ ground truth and the black ‘x’ is the ${\text{SpO}}_{{\text{2}}}$ level that the DST algorithm calculates. The ${\text{SpO}}_{{\text{2}}}$ ground truth for both subplots was 97.5%

The DST algorithm employs Adaptive Noise Cancellation (ANC) filters [11, 12] to remove noise, and provide a “clean” ${\text{SpO}}_{{\text{2}}}$. For each arbitrary ‘r’ (corresponding to an ${\text{SpO}}_{{\text{2}}}$ level between 50 and 100%), the reference signal and the infrared signal are fed into an ANC filter which identifies and removes frequency components which are in common between the two signals [2]. The power of the signal collected at the output of the ANC is calculated for each reference signal. A “DST plot” is generated, with the ${\text{SpO}}_{{\text{2}}}$ values used to generate the reference signals on the abscissa, and the power at the output of the ANC for each reference signal on the ordinate. Figure 6 shows the DST plots for clean (Fig. 6a) and noise-contaminated (Fig. 6b) synthetic PPG signals (for the way synthetic PPG signals were generated, see Sect. 3.1). In the case of a clean PPG signal, the DST plot shows only one peak. Its location corresponds to the ${\text{SpO}}_{{\text{2}}}$ level estimate (Fig. 6a). The ${\text{SpO}}_{{\text{2}}}$ level calculated by the DST algorithm for this synthetic PPG signals (the black ‘x’ on Fig. 6a) matches the ${\text{SpO}}_{{\text{2}}}$ level for which it was created (red circle on Fig. 6a). For the noisy signal, two distinct peaks will typically appear, as shown in Fig. 6b. One peak corresponds to the true ${\text{SpO}}_{{\text{2}}}$ level and the other peak is generated by noise. The right-most peak is considered to correspond the true ${\text{SpO}}_{{\text{2}}}$ level. The ${\text{SpO}}_{{\text{2}}}$ level calculated by the DST algorithm in the example used for Fig. 6b (the black ‘x’) deviates slightly from the ground truth (red circle). This difference is attributed to noise. The signal to noise ratio (defined in Eq. (10)) in this example was 0 dB, which is quite low.

## Generation of PPG and ECG signals

### Synthetic data generation

In order to study behavior, performance, and tradeoffs in the design of ${\text{SpO}}_{{\text{2}}}$ estimators, we developed a synthetic PPG signal generator. We modeled the PPG red (Eq. (6a)) and infrared (Eq. (6b)) waveforms as the sum of a constant DC component and an AC component. The AC component is the sum of four sinusoids of different amplitude^3^$A_j$ (j = 1, 2, 3, 4) (Eq. (7)). The first is at a frequency ranging from 0.5 to 3.5 Hz (corresponding to the subject’s heart rate and serving as the fundamental frequency). The three other sinusoids are its second, third, and fourth harmonics.

6a $$\begin{aligned} Red (t) = DC_{red} + AC_{red} \end{aligned}$$

6b $$\begin{aligned} Infrared (t) = DC_{infrared} + AC_{infrared} \end{aligned}$$

7 $$\begin{aligned} AC_{red} (t) = \sum _{i=1}^{4} -A_j \sin (2\pi \times j\times f_0\times t) \end{aligned}$$

where $$A_1=1.242 \times 10^{-3}$$, $$(A_2=0.835 \times 10^{-3}$$, $$A_3=1.899 \times 10^{-4}$$, and $$A_4=0.786 \times 10^{-4}$$.

**Fig. 7:**
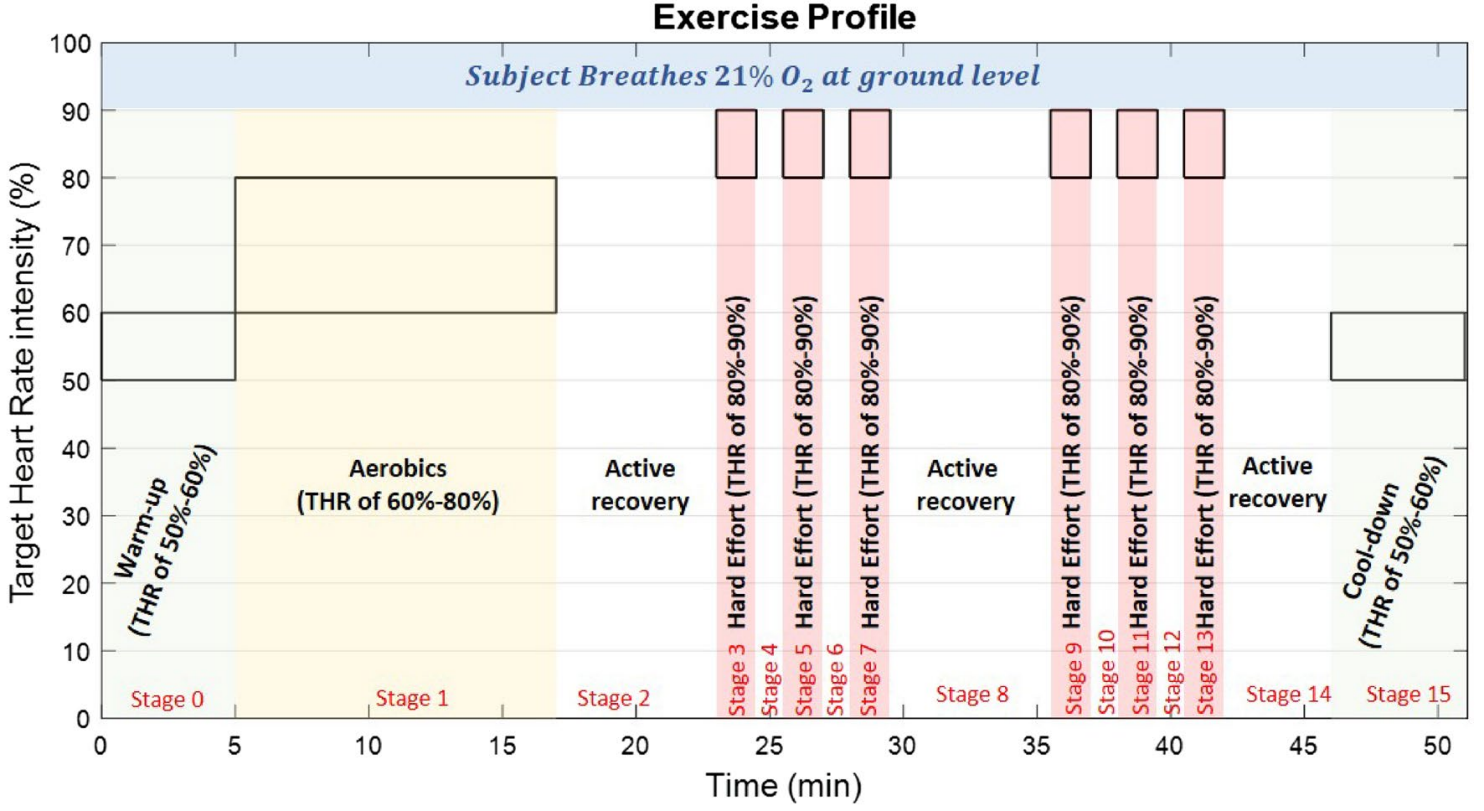
Illustration of the phases of the exercise experimental profile along with Target Heart Rate (THR) intensity. Black boxes indicate the range of THR in each exercise phase

${\text{SpO}}_{{\text{2}}}$ levels and the optical density ratio ‘r’ are related by the calibration curve (see Eq. (2)). In simulations on synthetic PPG signals, we varied the ratio of red-to-infrared signals in order to get the desired ${\text{SpO}}_{{\text{2}}}$ level. The optical density ratio ‘r’ is defined as

8 $$r = \frac{{\left( {\frac{{AC}}{{DC}}} \right)red}}{{\left( {\frac{{AC}}{{DC}}} \right)rinfrared}} = \frac{{Normalized_{{red}} }}{{Normalized_{{infrared}} }},$$

Hence,

9 $$\begin{aligned} AC_{infrared}= \frac{DC_{infrared}}{DC_{red}\times r} \times AC_{red} . \end{aligned}$$

The ECG signal is generally composed of P, QRS, T, and U waves [24]. Islam et al. [25] modeled a synthetic clean ECG waveforms by assuming that the QRS, Q, and S portions of the ECG signal can be represented by triangular waveforms, and the P, T, and U portions can be represented by the positive half period of a sinusoidal waveform. Their model was used in this study to generate the synthetic ECG waveforms [26].

### Human subject data (Real PPG)

We have used human subject data from an experiment conducted in 2018–2019 by the United States Naval Air Warfare Center Aircraft Division (NAWCAD). Data were collected from fourteen (14) test subjects who were briefed and provided informed consent. All subjects were volunteers, military or civil-service personnel in the employ of the US federal government. All subjects were non-smokers and were reviewed by a medical monitor to ensure that they were physically fit to participate.^4^

Each subject was exposed to the following profile of activities (Fig. 7).

1. Warm-up—a warm-up and stretching session, designed to raise and maintain Target Heart Rate (THR)^5^ intensity of 50–60% (approximately 5 min).
2. Aerobic—run on treadmill/bike session to raise and maintain THR of 60–80% (approximately 12 min), followed by active recovery (approximately 6 min), designed to decrease the heart rate by a reduced-intensity jog/walk or cycling activity.
3. Anaerobic—two sessions, each consisting of three 90-s periods of strenuous effort (treadmill/bike) designed to raise and maintain THR of 80–90%, separated from each other by one (1) minute of active recovery, designed to decrease heart rate by a reduced-intensity jog/walk or cycling; the two sessions were separated from each other by a period of active recovery (approximately 6 min). Next, the subject undertook a period of active recovery (around 4 min). 4. Cool-down—a cool-down and stretching session to maintain a THR of 50–60% (about 5 min).

Figure 7 shows all stages of this regime, marked stage 0 to stage 15. During the experimental runs, signals were recorded from a prototype dual pulse oximeter system with an accelerometer worn on the arm developed by Athena GTX (Holistic Modular Aircrew Physiologic Status (HMAPS) Monitoring System), and ECG electrodes affixed to the chest. Additionally, a Nonin 8000*R* reflectance pulse oximetry sensor was mounted on the subject’s temple and connected by wires to the Nonin Wrist-Ox 3150 for data processing. The 8000*R* sensor was mounted on the subject’s temple in a solid and stable manner that minimized sensor movements or dislocations on the temple surface even during strenuous exercise. ${\text{SpO}}_{{\text{2}}}$ levels calculated by the temple Nonin 8000*R* sensor were considered the “ground truth” due to the high accuracy of this sensor.^6^ Performance of the arm-mounted oximeters was assessed with respect to the readings of the temple-mounted 8000*R* sensor (Sect. 4.2).

## Impact of a heart-rate tuned comb filter on ${\text{SpO}}_{{\text{2}}}$ measurement performance

### ${\text{SpO}}_{{\text{2}}}$ level calculations on synthetic data

We set out to assess the effect of the heart-rate tuned comb filter on accuracy of calculated ${\text{SpO}}_{{\text{2}}}$ levels.^7^ To this end, we employed the “Red over Infrared” approach and the DST-based algorithm on a 10-s long synthetic PPG waveforms, with and without pre-filtering of the PPG signal by a comb filter. ${\text{SpO}}_{{\text{2}}}$ level and heart rate were set to 95% and 60 bpm, respectively. We modeled the motion artifact noise, N, as a bandpass (0.5–5 Hz) filtered AWGN (as was done in [28]), added to the clean PPG signal, S. The signal-to-noise ratio (SNR) is

10 $$\begin{aligned} SNR= \frac{Var(S)}{Var(N)} . \end{aligned}$$

**Fig. 8:**
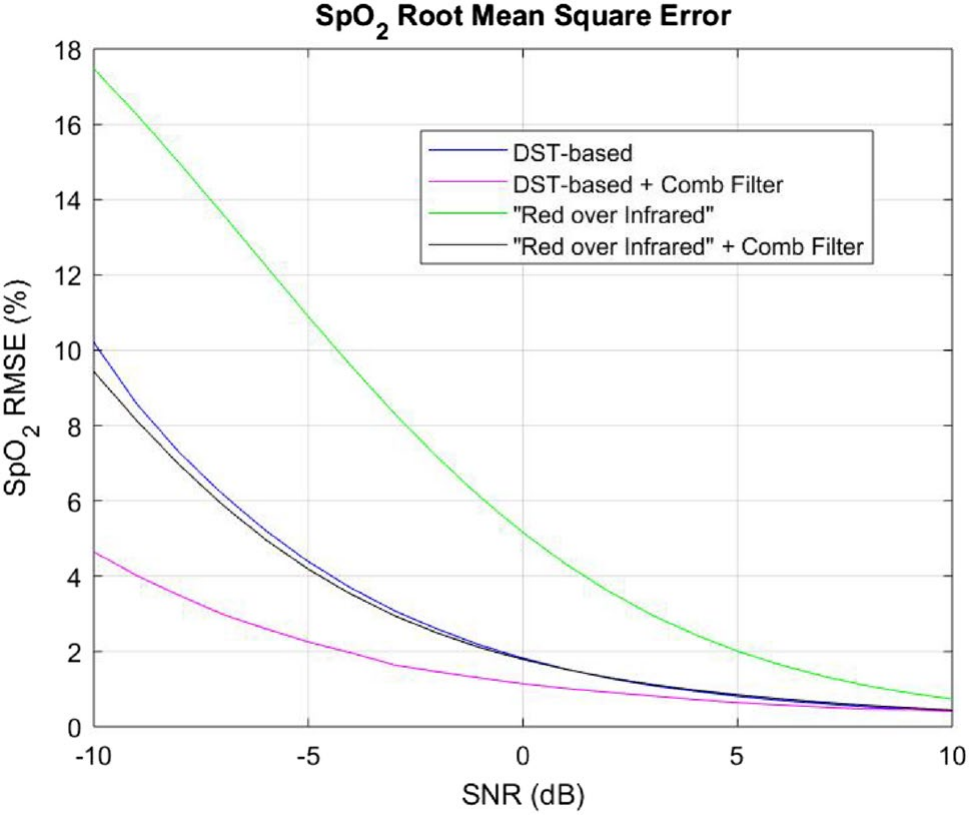
${\text{SpO}}_{{\text{2}}}$ Root Mean Square Error using “Red over Infrared” approach and the DST-based algorithm, with and without a comb filter

**Fig. 9:**
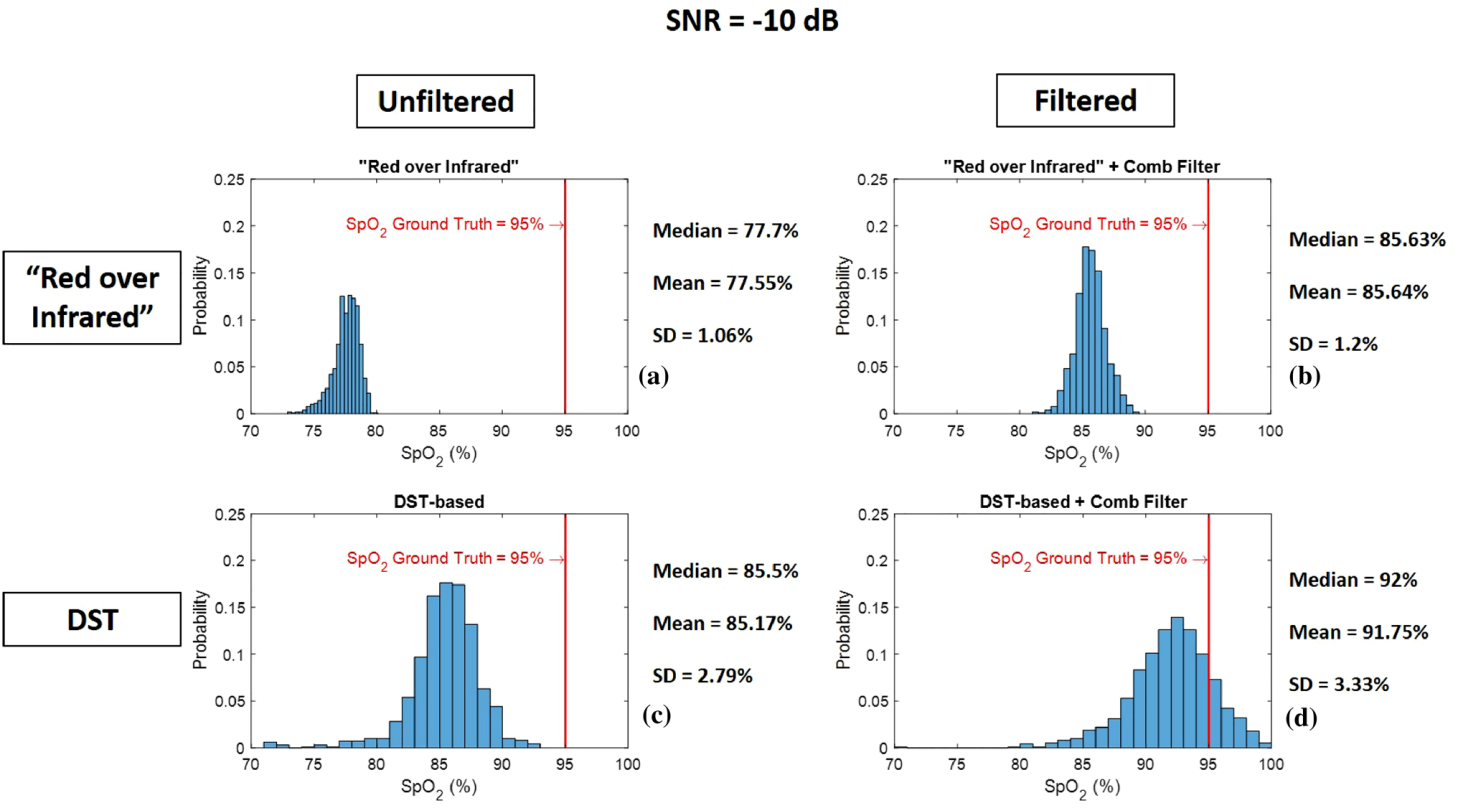
Histogram of 1000 ${\text{SpO}}_{{\text{2}}}$ levels calculated from red and infrared PPG signal with SNR = −10 dB using **a** “Red over Infrared” approach—**b** “Red over Infrared” approach preceded by a heart-rate tuned comb filter—**c** DST-based algorithm—**d** DST-based algorithm preceded by a heart-rate tuned comb filter

**Fig. 10:**
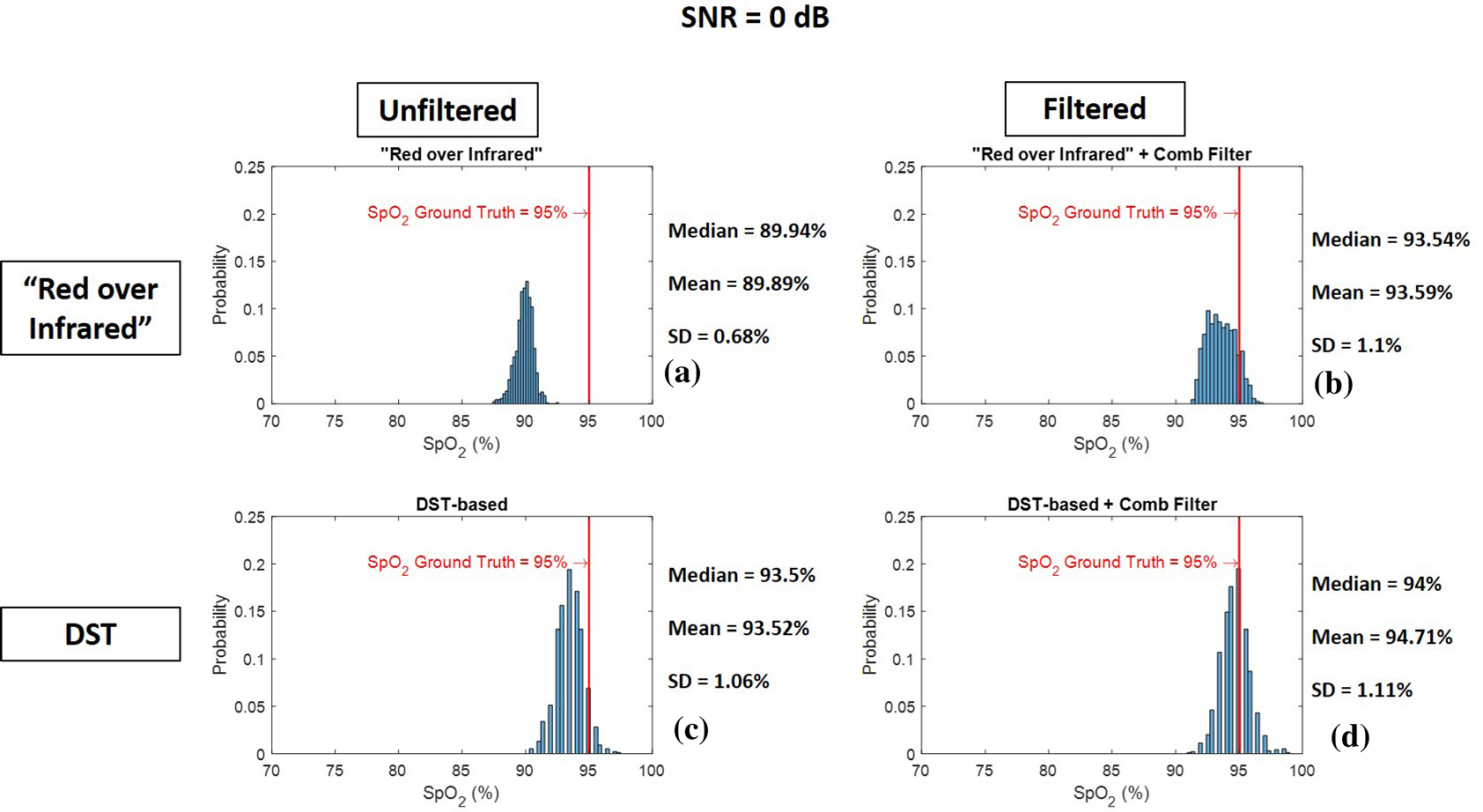
Histogram of 1000 ${\text{SpO}}_{{\text{2}}}$ levels calculated from red and infrared PPG signal with SNR = 0 dB using **a** “Red over Infrared” approach—**b** “Red over Infrared” approach preceded by a heart-rate tuned comb filter—**c** DST-based algorithm—**d** DST-based algorithm preceded by a heart-rate tuned comb filter

**Fig. 11:**
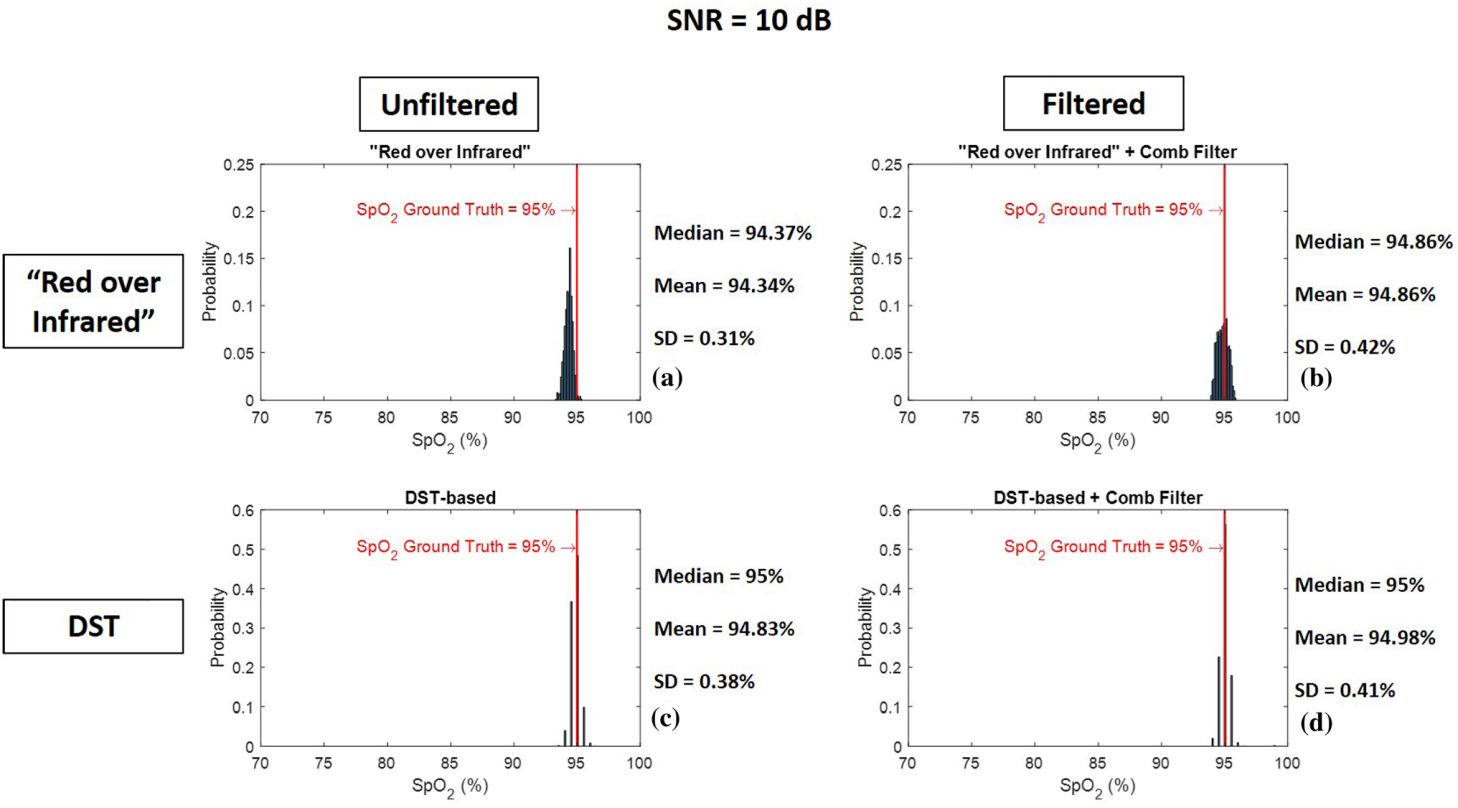
Histogram of 1000 ${\text{SpO}}_{{\text{2}}}$ levels calculated from red and infrared PPG signal with SNR = 10 dB using **a** “Red over Infrared” approach—**b** “Red over Infrared” approach preceded by a heart-rate tuned comb filter—**c** DST-based algorithm—**d** DST-based algorithm preceded by a heart-rate tuned comb filter

On Fig. 8 we show the Root Mean Square Error (RMSE) of ${\text{SpO}}_{{\text{2}}}$ levels calculated from synthetic PPG signals for an SNR ranging from −10 to +10 dB. We used the “Red over Infrared” approach and the DST-based algorithm, both before and after processing the synthetic PPG signals with the heart-rate tuned comb filter. The RMSE is defined as

11 $$\begin{aligned} RMSE(i)= \sqrt{\frac{1}{P} \sum _{p=1}^{P} {({ {\text{SpO}}_{{\text{2}}} }_{cal.}(p) - { {\text{SpO}}_{{\text{2}}} }_{g.t.})}^2} , \end{aligned}$$

where ‘${ {\text{SpO}}_{{\text{2}}} }_{cal.}(p)$’ is one value (out of the 1000 realizations) of the ${\text{SpO}}_{{\text{2}}}$ level calculated using one of the tested methods (“Red over Infrared” or DST-based algorithm, with and without comb filtering); ‘${ {\text{SpO}}_{{\text{2}}} }_{g.t.}$’ is the ground truth ${\text{SpO}}_{{\text{2}}}$ value; ‘*P*’ is the number of ${\text{SpO}}_{{\text{2}}}$ levels calculated for each SNR, and ‘*i*’ the SNR value at which the RMSE is calculated. In our study, ${ {\text{SpO}}_{{\text{2}}} }_{g.t.}=95\%, P=1000$ realizations, and $i = -10, -9, \ldots , -1, 0, 1, \ldots , 9, 10$ (dB).

The main conclusion from Fig. 8 is that the unfiltered “Red over Infrared” approach is inferior to the other approaches (namely filtered “Red over Infrared” and DST-based algorithm (filtered or unfiltered)). The filtered “Red over Infrared” approach and the unfiltered DST-based algorithm are comparable. The best performance was obtained by the filtered DST-based algorithm.

Figures 9, 10, and 11 are the histograms of ${\text{SpO}}_{{\text{2}}}$ levels (1000 ${\text{SpO}}_{{\text{2}}}$ calculations each) calculated using “Red over Infrared” approach and the DST-based algorithm, both with and without processing the synthetic PPG signals with the heart-rate tuned comb filter. We show results for a SNR of −10 dB (Fig. 9), 0 dB (Fig. 10), and 10 dB (Fig. 11). The histograms get closer to the ${\text{SpO}}_{{\text{2}}}$ ground truth (${\text{SpO}}_{{\text{2}}}$ of 95%) when the synthetic PPG signals are preprocessed by the heart-rate tuned comb filter for both ${\text{SpO}}_{{\text{2}}}$ calculation methods (“Red over Infrared” and the DST). We conclude that (1) the use of a heart-rate tuned comb filter has improved the performance of both methods (“Red over Infrared” and the DST), and (2) the best performance is obtained with the DST-based algorithm preceded by a heart-rate tuned comb filter. Next best is the “Red over Infrared” approach with comb filtering and unfiltered DST-based algorithm (${\text{SpO}}_{{\text{2}}}$ levels estimated with these two techniques are comparable); the worst performance was the unfiltered “Red over Infrared” approach.

**Table 2:** Detailed description on all traces shown in Fig. 8

	Trace label	Trace color	Trace name	Description
Figure 12a	$a_1$	Blue	X	x-axis accelerometer
	$a_2$	Red	Y	y-axis accelerometer
	$a_3$	Orange	Z	z-axis accelerometer
Figure 12b	$b_1$	Light blue	Nonin ${\text{SpO}}_{{\text{2}}}$	${\text{SpO}}_{{\text{2}}}$ levels calculated by Nonin 8000*R* sensor
	$b_2$	Green (top curve)	${\text{SpO}}_{{\text{2}}}$ “Red over Infrared”	${\text{SpO}}_{{\text{2}}}$ levels calculated using “Red over Infrared” approach before comb filter
	$b_3$	Black (top curve)	${\text{SpO}}_{{\text{2}}}$ “Red over Infrared” + Comb Filter	${\text{SpO}}_{{\text{2}}}$ levels calculated using “Red over Infrared” approach after comb filter
	$b_4$	Green (bottom curve)	${\text{SpO}}_{{\text{2}}}$ Difference between Nonin and “Red over Infrared”	${\text{SpO}}_{{\text{2}}}$ Difference between levels calculated by Nonin 8000*R* sensor and “Red over Infrared” approach before comb filter
	$b_5$	Black (bottom curve)	${\text{SpO}}_{{\text{2}}}$ Difference between Nonin and “Red over Infrared” + Comb Filter	${\text{SpO}}_{{\text{2}}}$ Difference between levels calculated by Nonin 8000*R* sensor and “Red over Infrared” approach after comb filter
Figure 12c	$c_1$	Light blue	Nonin ${\text{SpO}}_{{\text{2}}}$	${\text{SpO}}_{{\text{2}}}$ levels calculated by Nonin 8000*R* sensor
	$c_2$	Dark blue (top curve)	${\text{SpO}}_{{\text{2}}}$ DST-based	${\text{SpO}}_{{\text{2}}}$ levels calculated using DST-based algorithm before comb filter
	$c_3$	Magenta (top curve)	${\text{SpO}}_{{\text{2}}}$ DST-based + Comb Filter	${\text{SpO}}_{{\text{2}}}$ levels calculated using DST-based algorithm after comb filter
	$c_4$	Dark blue (bottom curve)	${\text{SpO}}_{{\text{2}}}$ Difference between Nonin and DST-based	${\text{SpO}}_{{\text{2}}}$ Difference between levels calculated by Nonin 8000*R* sensor and DST-based algorithm before comb filter
	$c_5$	Magenta (bottom curve)	${\text{SpO}}_{{\text{2}}}$ Difference between Nonin and DST-based + Comb Filter	${\text{SpO}}_{{\text{2}}}$ Difference between levels calculated by Nonin 8000*R* sensor and DST-based algorithm after comb filter
Figure 12d	$d_1$	Light blue	Nonin ${\text{SpO}}_{{\text{2}}}$	${\text{SpO}}_{{\text{2}}}$ levels calculated by Nonin 8000*R* sensor
	$d_2$	Magenta (top curve)	${\text{SpO}}_{{\text{2}}}$ DST-based + Comb Filter	${\text{SpO}}_{{\text{2}}}$ levels calculated using DST-based algorithm after comb filter.
	$d_3$	Black (top curve)	${\text{SpO}}_{{\text{2}}}$ “Red over Infrared” + Comb Filter	${\text{SpO}}_{{\text{2}}}$ levels calculated using “Red over Infrared” approach after comb filter
	$d_4$	Magenta (bottom curve)	${\text{SpO}}_{{\text{2}}}$ Difference between Nonin and DST-based + Comb Filter	${\text{SpO}}_{{\text{2}}}$ Difference between levels calculated by Nonin 8000*R* sensor and DST-based algorithm after comb filter
	$d_5$	Black (bottom curve)	${\text{SpO}}_{{\text{2}}}$ Difference between Nonin and “Red over Infrared” + Comb Filter	${\text{SpO}}_{{\text{2}}}$ Difference between levels calculated by Nonin 8000*R* sensor and “Red over Infrared” approach after comb filter

**Fig. 12:**
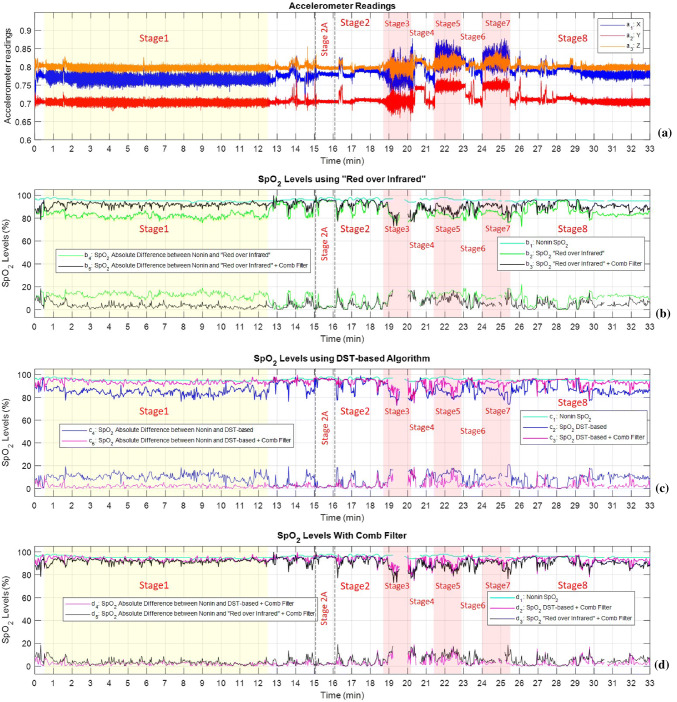
${\text{SpO}}_{{\text{2}}}$ level calculations for an exercising subject on stages 1 to 8 (see Sect. 3.2 and Fig. 7). We used the “Red over Infrared” approach and DST-based algorithm with and without preprocessing of the PPG signals with the heart-rate tuned comb filter. ${\text{SpO}}_{{\text{2}}}$ levels calculated using the abovementioned algorithms is compared to the ${\text{SpO}}_{{\text{2}}}$ levels calculated by Nonin 8000*R* sensor. The legends are fully explained in Table 2

**Table 3:** Overall Mean and standard deviation (SD) of the ${\text{SpO}}_{{\text{2}}}$ error calculated for “Red over Infrared” approach and DST-based algorithm with and without comb filtering for all 14 exercise subjects

	R/IR		R/IR + comb		DST		DST + comb	
	Mean	SD	Mean	SD	Mean	SD	Mean	SD
Stage 1	14.03	1.54	9.71	1.72	11.34	1.52	7.55	1.64
Stage 2	7.05	1.37	4.74	1.15	4.76	1.1	3.34	0.93
Stage 3	10.49	0.87	7.59	1.04	8.82	1.02	5.64	1.04
Stage 4	9.82	1.56	6.18	1.29	5.85	1.23	4.07	1.13
Stage 5	11.6	1.11	7.49	1.09	8.32	1.03	5.01	0.99
Stage 6	9.01	1.42	4.86	1.11	5.52	1.19	3.09	0.9
Stage 7	11.2	0.96	7.72	1.49	7.91	1.02	4.46	1.03
Stage 8	8.14	1.49	5.03	1.02	6.1	1.58	3.45	0.99

**Table 4:** Tested hypotheses and results

	Is the ${\text{SpO}}_{{\text{2}}}$ mean absolute error of ...		${\text{SpO}}_{{\text{2}}}$ mean absolute error of ...?		
Test I:	“Red over Infrared” approach (R/IR) *without* comb filter ($\mu _1$)	Greater than	“Red over Infrared” approach (R/IR) *with* comb filter ($\mu _2$)	$H_0$: $\mu _1$−$\mu _2 = 0$ $H_a$: $\mu _1$−$\mu _2$ > 0	Yes. (test I, Tables 5, 7, and 8 in Appendix)
Test II:	“Red over Infrared” approach (R/IR) *without* comb filter ($\mu _1$)	Greater than	“DST-based” algorithm (DST) *without* comb filter ($\mu _3$)	$H_0$: $\mu _1$−$\mu _3 = 0$ $H_a$: $\mu _1$−$\mu _3$ > 0	Yes. (test II, Tables 5, 7, and 8 in Appendix)
Test III-a:	“Red over Infrared” approach (R/IR) *with* comb filter ($\mu _2$)	Different than	“DST-based” algorithm (DST) *without* comb filter ($\mu _3$)	$H_0$: $\mu _2$−$\mu _3 = 0$ $H_a$: $\mu _2$−$\mu _3$ $\ne$ 0	No^a^. (test III-a, Tables 5, 7, and 8 in Appendix)
Test III-b:	“DST-based” algorithm (DST) *without* comb filter ($\mu _3$)	Greater than	“Red over Infrared” approach (R/IR) *with* comb filter ($\mu _2$)	$H_0$: $\mu _3$−$\mu _2 = 0$ $H_a$: $\mu _3$−$\mu _2$ > 0	No^b^. (test III-b, Tables 5, 7, and 8 in Appendix)
Test IV:	“Red over Infrared” approach (R/IR) *with* comb filter ($\mu _2$)	Greater than	“DST-based” algorithm (DST) *with* comb filter ($\mu _4$)	$H_0$: $\mu _2$−$\mu _4 = 0$ $H_a$: $\mu _2$−$\mu _4$ > 0	Yes. (test IV, Tables 5, 7, and 8 in Appendix)
Test V:	“DST-based” algorithm (DST) *without* comb filter ($\mu _3$)	Greater than	“DST-based” algorithm (DST) *with* comb filter ($\mu _4$)	$H_0$: $\mu _3$−$\mu _4 = 0$ $H_a$: $\mu _3$−$\mu _4$ > 0	Yes. (test V, Tables 5, 7, and 8 in Appendix)

^a^For calibration curve (2) the answers are:

For stage 1—No, stage 2—No, stage 3—Yes, stage 4—No, stage 5—No, stage 6—No, stage 7—No, stage 8—No

Overall answer: No

For calibration curve (3) the answer is No for all stages

For calibration curve (4) the answer is No for all stages

^b^For calibration curve (2) the answers are:

For stage 1—Yes, stage 2—No, stage 3—Yes, stage 4—No, stage 5—No, stage 6—No, stage 7— No, stage 8—No

Overall answer: No

For calibration curve (3) the answers are:

For stage 1—Yes, stage 2—No, stage 3—No, stage 4—No, stage 5—No, stage 6—No, stage 7—No, stage 8—No

Overall answer: No

For calibration curve (4) the answers are:

For stage 1—Yes, stage 2—No, stage 3——No, stage 4—No, stage 5—No, stage 6—No, stage 7—No, stage 8—No

Overall answer: No

**Table 5:** p values of all six tests

p value (significance level was *α* = 0.01)						
	Test I	Test II	Test III-a	Test III-b	Test IV	Test V
Stage 1	< 0.00001	0.00023	0.01975	0.00987	0.00237	< 0.0001
Stage 2	0.00016	0.00015	0.01004	0.48161	0.00181	0.00136
Stage 3	< 0.00001	0.00022	0.00753	0.00377	0.00013	< 0.00001
Stage 4	< 0.00001	< 0.00001	0.25116	0.25025	0.00025	0.00077
Stage 5	< 0.00001	< 0.00001	0.05893	0.02941	< 0.0001	< 0.00001
Stage 6	< 0.00001	< 0.00001	0.15245	0.07654	0.00024	<0.0001
Stage 7	< 0.00001	< 0.00001	0.35143	0.35009	< 0.00001	< 0.00001
Stage 8	0.000011	0.00192	0.02643	0.26483	0.00056	< 0.0001

### ${\text{SpO}}_{{\text{2}}}$ level calculations on experimental data

#### Data analysis using the standard model calibration curve equation (2)

We tested the “Red over Infrared” approach and the DST-based algorithm on data collected from human subjects, with and without comb filtering. Data were collected from fourteen (14) human subjects for the regime described in Sect. 3.2 and Fig. 7. Every 2 s, the preceding 10-s long data segment was processed. We show the results of one of the fourteen (1 of 14) subjects on Fig. 12 (we covered the time period from 5 to 35 min of the exercise profile, corresponding to stages 1 to 8 in Fig. 7). A detailed description of the traces in Fig. 12 is provided in Table 2.^8^ In all cases, the standard against which the various methods were assessed was the readings of the Nonin 8000R sensor (light blue trace in Fig. 12b, c, d).

The gaps in ${\text{SpO}}_{{\text{2}}}$ curves on Fig. 12 (for example: In subplots b, c, and d from 19.5 to 20 min for the “Red over Infrared” approach and the DST-based algorithm, and from around time 20.5 min for Nonin 8000*R* sensor) indicate a failure in calculating the ${\text{SpO}}_{{\text{2}}}$ level caused by physical loss of the PPG signal. We skipped these gaps in our analysis.

Examination of Fig. 12 suggests that the addition of a comb filter improved performance for both oximeters based on R/IR and oximeters based on DST algorithm (Fig. 12 second and third trace). Also, performance of oximeters employing R/IR + comb filter was very close to performance of oximeters employing DST + comb filter (Fig. 12 fourth trace).

Table 3 provides the overall mean and standard deviation of the ${\text{SpO}}_{{\text{2}}}$ error for all fourteen (14) subjects we studied in all eight (8) exercise profile stages (stages 1 to 8 in Fig. 7). The ${\text{SpO}}_{{\text{2}}}$ error is defined as absolute difference between ${\text{SpO}}_{{\text{2}}}$ levels measured by Nonin 8000*R* sensor sensor mounted on the subject’s temple (the standard) and ${\text{SpO}}_{{\text{2}}}$ levels calculated by the two algorithms. We performed six (6) two-samples t significance tests with level of significance $\alpha$=0.01. The tested hypotheses and their corresponding p-values are shown in Tables 4 and 5, respectively.

We concluded from Tables 4 and 5 the following:

- The mean absolute error using R/IR was larger than the mean absolute error using R/IR + Comb in all eight (8) stages;
- The mean absolute error using R/IR was larger than the mean absolute error using DST in all eight (8) stages;
- The mean absolute error using R/IR + Comb was larger than the mean absolute error using DST + Comb in all eight (8) stages;
- The mean absolute error using DST was larger than the mean absolute error using DST + Comb in all eight (8) stages.

Additionally, we concluded from Tables 4 and 5 (tests III-a and III-b) that the mean absolute errors using R/IR + Comb and using DST are comparable during most stages.

#### Data analysis using alternate calibration curves eqs. (3) and (4)

Appendix 1 replicates Table 5 (p-values) for the alternate calibration curves (3) [Lambert-Beer calibration curve] Table 7 in Appendix and (4) [underestimation calibration curve] Table 8 in Appendix. These tables support the conclusion in Table 4 for the alternate calibration curves as well (see Table 4 footnotes a and b). The relative performance of the ${\text{SpO}}_{{\text{2}}}$ calculation techniques and impact of the comb filter are qualitatively the same, regardless of the specific calibration curve (be it (2), (3), or (4))

### Computational complexity

#### Red over infrared approach

For red and infrared signals of length L (L sampled values) the number of operations required for a typical “Red over Infrared” calculation is 2L multiplications, 2(L-1) additions, three divisions and two square root operations. For each L (in our case in the thousands), the required computation time is proportional to L.

**Fig. 13:**
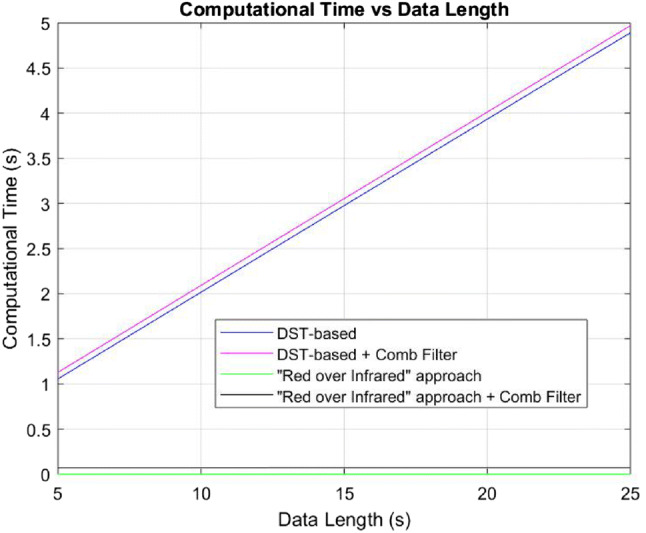
Comparison of computational time of the “Red over Infrared” approach and the DST-based Algorithm with and without comb filtering

#### DST algorithm

At every time instance, the DST algorithm generates a family of M reference signals (in our study we used M=101 reference signal for each optical density ratio of ${\text{SpO}}_{{\text{2}}}$ level, ranging from 50% to 100% with an increment of 0.5%). For each one of the M ${\text{SpO}}_{{\text{2}}}$ levels, the DST algorithm uses an ANC filter and additional computations to create one point on the DST graph of power vs. ${\text{SpO}}_{{\text{2}}}$ level (e.g. Fig. 6b in this paper and Fig. 3 in [2]). For the ANC we have used the QR-decomposition-based least-squares lattice (QRD-LSL) adaptive filter algorithm, on account of its relative computational efficiency. The QRD-LSL requires O(R) operations per time instance (we use L instances for block of data), where R is the number of taps in the adaptive filter [30]. The computational times (in seconds) of “Red over Infrared” approach and the DST-based algorithm (with and without comb filtering) are shown on the ordinate of Fig. 13. The abscissa of Fig. 13 is the red and infrared signals’ length in second. On Table 6 we show the computational time and the ${\text{SpO}}_{{\text{2}}}$ RMSE (in %) calculated on a 10-s long synthetic PPG signals. We use the “Red over Infrared” approach and the DST-based algorithm, both before and after comb filtering in each case, for different SNR values (−10 dB, 0 dB, and 10 dB).^9^ Results presented in Fig. 13 and Table 6 indicate that the computational costs of the DST-based algorithm are considerably larger than the “Red over Infrared” approach. The effects of adding the comb filter are much smaller.

### Implementation

The comb filter could be integrated into commercial systems due to the light hardware and computational requirements. We demonstrated this point by implementing the comb filter on an ARM Cortex-M4 based processor (model MK70FN1M0VMJ12).

**Table 6:** Computational time and ${\text{SpO}}_{{\text{2}}}$ RMSE calculated on a 10-s long PPG signals using “Red over Infrared” approach and DST-based algorithm before and after comb filtering for a SNR of −10dB, 0dB, and 10dB

	${\text{SpO}}_{{\text{2}}}$ RMSE (%)			Computational time (second)
	SNR = −10 dB	SNR = 0 dB	SNR = 10 dB	
”Red over infrared”	17.4811	5.1564	0.7301	$2.463\times 10^{-5}$
”Red over infrared” + comb	9.4388	1.7844	0.4425	$7.248\times 10^{-2}$
DST-based	10.2173	1.8191	0.4135	2.017
DST-based + comb	4.6482	1.1431	0.4056	2.092

## Discussion and conclusion

Preprocessing PPG signals with a heart-rate tuned comb filter improved the performance of the two tested ${\text{SpO}}_{{\text{2}}}$ calculation algorithms (namely, “Red over Infrared” approach and DST-based algorithm). We tested both algorithms on synthetic and experimental data.

- The most accurate technique was the filtered DST-based algorithm. At very low signal to noise (SNR) environments the filtered DST-based algorithm performed somewhat better on synthetic data compared to the other methods (up to 6% improvement in accuracy at minus 10 dB SNR over the unfiltered DST algorithm and the filtered “Red over Infrared” approach). However, this technique was costly in computations.
- Next best were the filtered “Red over Infrared” approach and the unfiltered DST-based algorithm, which provided similar accuracies. However, the DST-based algorithm was much costlier in computations compared to the filtered “Red over Infrared” approach.
- The least accurate performance was of the unfiltered “Red over Infrared” approach.

The overall conclusion is that if an uninterrupted high-quality heart rate measurement is available, then the pre-filtered “Red over Infrared” approach (using a heart-rate tuned comb filter) provides a preferred tradeoff between ${\text{SpO}}_{{\text{2}}}$-level accuracy and computational complexity.

## Limitations

1. In this study ${\text{SpO}}_{{\text{2}}}$ ground truth was considered the levels calculated using the highly-accurate Nonin 8000*R* sensor [29], mounted on the subject’s temple. It may have been preferable to draw blood samples from the subjects and measure arterial oxygen saturation directly. However, this invasive procedure was not permitted under the IRB-approved experimental protocol.
2. All fourteen (14) tested datasets were collected from healthy and physically fit subjects. Therefore, the effect of irregular heart rhythm on ${\text{SpO}}_{{\text{2}}}$ level calculation using our methods was not studied.
3. All fourteen (14) datasets available from the experiment were processed. We did not exclude any sample based on its corresponding ECG signal quality or other considerations. In this study, the heart rate derived from measurements by the Nonin sensor was not used in our calculation, nor investigated further.

## Appendix 1: p values for the two alternate calibration curves

We re-calculated the p values of Table 5 for the “Lambert-Beer curve” (Eq. (3)), and the “underestimation calibration curve” (Eq. (4)). We show the p-values of the tested hypotheses in Table 7 in Appendix for the “Lambert–Beer curve” and Table 8 in Appendix for the “underestimation calibration curve.” They support the conclusions provided in Table 4.

**Table 7:** p values of all six tests for “Lambert–Beer calibration curve” (Eq. (3))

p value (significance level was $\alpha$ = 0.01)						
	Test I	Test II	Test III-a	Test III-b	Test IV	Test V
Stage 1	< 0.00001	0.00015	0.01147	0.00573	0.00066	< 0.00001
Stage 2	< 0.0001	< 0.0001	0.96102	0.48083	0.00032	0.00032
Stage 3	< 0.00001	< 0.0001	0.07928	0.03958	< 0.0001	< 0.00001
Stage 4	< 0.00001	< 0.00001	0.51485	0.25760	< 0.0001	< 0.0001
Stage 5	< 0.00001	< 0.00001	0.10969	0.05477	< 0.00001	< 0.00001
Stage 6	< 0.00001	< 0.00001	0.26849	0.13412	< 0.0001	< 0.00001
Stage 7	< 0.00001	< 0.00001	0.16402	0.08195	< 0.00001	< 0.00001
Stage 8	< 0.00001	0.00024	0.28919	0.14459	< 0.0001	< 0.0001

**Table 8:** p values of all six tests for “underestimation calibration curve” (Eq. (4))

p value (significance level was $\alpha$ = 0.01)						
	Test I	Test II	Test III-a	Test III-b	Test IV	Test V
Stage 1	< 0.00001	0.00046	0.01024	0.00511	0.00142	< 0.00001
Stage 2	0.00012	0.00010	0.98250	0.49139	0.00041	0.00036
Stage 3	< 0.00001	< 0.0001	0.07269	0.03629	< 0.0001	< 0.00001
Stage 4	< 0.00001	< 0.00001	0.49433	0.24723	< 0.0001	< 0.0001
Stage 5	< 0.00001	< 0.00001	0.15623	0.078109	< 0.00001	< 0.00001
Stage 6	< 0.00001	< 0.00001	0.28380	0.14178	< 0.0001	< 0.0001
Stage 7	< 0.00001	< 0.00001	0.18906	0.09453	< 0.00001	< 0.00001
Stage 8	< 0.0001	0.00038	0.24414	0.12205	< 0.0001	< 0.0001

## Footnotes

1 Details about the exercise profile are provided in Sect. 3.2. This waveform was obtained from stage 8 (active recovery stage).

2 The pulse oximeters reported upon in [15] were the following: Masimo Radical-7, Nihon Kohden OxyPal Neo, Nellcor N-600, and Philips Intellivue MP5.

3 The values of $A_j$s were empirically derived from clean PPG waveforms taken from human subjects at rest.

4 Study approved by Naval Air Warfare Center Aircraft Division IRB, protocol FWR21070114H, original approval date: 12 June 2017. Air Force Research Lab (AFRL) IRB protocols comply with DoD Directive 3216.02, Title 25, CFR 46, and are in compliance with the Declaration of Helsinki Revision 6, 2008.

5 The target Heart Rate (THR) is determine using the Karvonen formula [27] THR = (($HR_{max}$—$HR_{rest}$) $\times$ (%intensity)) + $HR_{rest}$, where $HR_{max} = 208$ – 0.7 $\times$ age [28].

6 In general, reflectance pulse oximetry, such as the method used by Nonin 8000*R* is known to be much less vulnerable to artifacts (including motion artifacts). The manufacturer reports that ${\text{SpO}}_{{\text{2}}}$ accuracy of the Model 8000*R* sensor was determined through an induced hypoxia study on healthy subjects over the range of 70% to 100% [29]. The resulting ${\text{SpO}}_{{\text{2}}}$ accuracy was ± 2$A_{rms}$ in the range 80–100% and ± 3$A_{rms}$ in the range 70–80%. ± 1$A_{rms}$ encompasses 68% of the population at zero bias.

7 In this section we have used calibration curve (2). We have repeated the calculation for calibration curves (3) and (4) and the trends and conclusion remain the same.

8 Data for all 14 subjects is available at [https://github.com/moshekam/PPG-Exercise-Experimental-Data.git].

9 The results were generated by MatLab R2018a on a personal computer, with an Intel Core$^{TM}$ i5-8500 CPU running at 3.00 GHz, 8GB RAM and Windows 10 operating system.
